# Identification of novel citrullinated autoantigens of synovium in rheumatoid arthritis using a proteomic approach

**DOI:** 10.1186/ar2085

**Published:** 2006-11-27

**Authors:** Kosuke Matsuo, Yang Xiang, Hiroshi Nakamura, Kayo Masuko, Kazuo Yudoh, Koji Noyori, Kusuki Nishioka, Tomoyuki Saito, Tomohiro Kato

**Affiliations:** 1 Department of Bioregulation & Proteomics, Institute of Medical Science, St. Marianna University School of Medicine, Sugao 2-16-1, Miyamae, Kawasaki, Kanagawa 216-8512, Japan; 2 Musculoskeletal Science, Yokohama City University Graduate School of Medicine, Fukuura3-9, Kanazawa, Yokohama, Kanagawa 236-0004, Japan; 3 Department of Frontier Medicine, Institute of Medical Science, St. Marianna University School of Medicine, Sugao 2-16-1, Miyamae, Kawasaki, Kanagawa 216-8512, Japan

## Abstract

Recently, autoantibodies to some citrullinated autoantigens have been reported to be specific for rheumatoid arthritis (RA). However, an entire profile of and autoimmunity of the citrullinated proteins have been poorly understood. To understand the profile, we examined citrullinated autoantigens by a proteomic approach and further investigated the significance of citrullination in antigenicity of one of the autoantigens. Specifically, we detected citrullinated autoantigens in synovial tissue of a patient with RA by two-dimensional electrophoresis and Western blotting by using pooled sera from five patients with RA and anti-citrulline antibodies. After identifying the detected autoantigens by mass spectrometry, we investigated the contribution of citrullination to autoantigenicity by using a recombinant protein with or without citrullination on one of the identified novel citrullinated autoantigens. As a result, we found 51 citrullinated protein spots. Thirty (58.8%) of these spots were autoantigenic. We identified 13 out of the 30 detected citrullinated autoantigenic proteins. They contained three fibrinogen derivatives and several novel citrullinated autoantigens (for example, asporin and F-actin capping protein α-1 subunit [CapZα-1]). We further analyzed the contribution of citrullination to autoantigenicity in one of the detected citrullinated autoantigens, CapZα-1. As a result, frequencies of autoantibodies to non-citrullinated CapZα-1 were 36.7% in the RA group tested, 10.7% in the osteoarthritis (OA) group, and 6.5% in healthy donors. On the other hand, those to citrullinated CapZα-1 were 53.3% in the RA group, 7.1% in the OA group, and 6.5% in the healthy donors. This shows that autoantigenicity of citrullinated or non-citrullinated CapZα-1 is relevant to RA. The antibody titers to the citrullinated CapZα-1 were significantly higher than those to the non-citrullinated CapZα-1 in 36.7% of patients; however, the other patients showed almost equal antibody titers to both citrullinated and non-citrullinated CapZα-1. Therefore, the autoantibodies would target citrulline-related and/or citrulline-unrelated epitope(s) of CapZα-1. In conclusion, we report a profile of citrullinated autoantigens for the first time. Even though citrullination is closely related to autoantigenicity, citrullination would not always produce autoantigenicity in RA. Citrullinated and non-citrullinated autoantigens/autoepitopes would have different pathological roles in RA.

## Introduction

Rheumatoid arthritis (RA) is one of the most prevalent rheumatic disorders and is characterized by chronic inflammation of multiple joints. It affects synovium, articular cartilage, and articular bones, which lead to destruction of the joints. Although the pathogenesis of RA is not fully understood, autoimmune reactions are suggested to play pathological roles in chronic synovitis. So far, a variety of candidate autoantigens such as rheumatoid factor, collagen type II, cartilage intermediate layer protein, YKL-39, and calpastatin have been suggested to induce cellular and/or humoral autoimmune responses in RA [1-5]. Autoantibodies directed to proteins with a non-standard amino acid of citrulline, produced by post-translational modification of arginine, have been found to be RA-specific [6,7]. Filaggrin is a typical example. In early studies, the autoantibodies to filaggrin, previously called 'anti-perinuclear factor antibodies' or 'anti-keratin antibodies,' were reported to be specific for RA. Later, citrullination was found to be essential for the autoantigenicity of filaggrin [6]. Quite recently, the anti-citrullinated protein antibodies have started to be measured using artificial cyclic citrullinated peptides (CCPs) as a clinical laboratory examination. The anti-CCP antibody was reported to have high predictive value for development of RA as well as high sensitivity and specificity for diagnosis of RA [5,8]. Since then, several autoantibodies against citrullinated proteins have been identified in RA. They include fibrin/fibrinogen [9], vimentin [10], and Epstein-Barr virus nuclear antigen-1 (EBVA-1) [11]. Concurrently, association of functional haplotypes of the gene encoding citrullinating enzyme of peptidylarginine deiminase-4 (PADI4) with susceptibility to RA was reported [12]. It was also reported that PADI4 affected levels of the antibody to citrullinated peptides in sera from patients with RA [12].

Pathologically, the antibodies to citrullinated proteins are expected to be produced in the synovial compartment [13] given that the anti-CCP antibodies constituted a higher proportion of immunoglobulin (Ig) G) in synovial fluid (SF) than that in serum of patients with RA [13,14] and given that B cells producing the anti-CCP antibodies have been isolated from RA synovium [14]. Furthermore, peptidylarginine deiminase (PAD) generates citrulline residues by deimination of arginine residues of proteins. Isoforms 2 and 4 of PAD were expressed in mononuclear cells isolated from SF [15]. These data suggest that presence of citrullinated proteins in the RA synovium causes antigen-driven maturation of B cells at the site of inflammation. However, it is poorly understood what kind of proteins are citrullinated and recognized as targets of autoantibodies in the synovial tissue of patients with RA. To answer these questions, comprehensive analysis of autoantigenic citrullinated proteins in RA would be needed. Based on this background, we performed a screening of autoantigenic citrullinated proteins in synovial tissue proteins from a patient with RA and evaluated the contribution of citrullination to autoantigenicity by using recombinant proteins.

## Materials and methods

### Patients and synovial tissues

Serum samples were obtained from 30 patients with RA (26 women, 4 men; ages 29 to 78 years, mean 60.1 years) and 28 patients with osteoarthritis (OA) (23 women, 5 men; ages 23 to 84 years, mean 64.4 years). The patients were diagnosed according to the respective classification criteria for each of the two diseases [16,17]. Serum samples from age- and gender-matched healthy donors were used as a control (27 women, 4 men; ages 32 to 80 years, mean 60.0 years). Non-RA rheumatological control samples were obtained from patients with systemic lupus erythematosus (SLE) (17 women, 2 men; ages 39 to 65 years, mean 49.7) who were diagnosed according to the published classification criteria [18]. Serum samples from age- and gender-matched health donors (21 women, 1 man; ages 32 to 64 years, mean 52.7 years) were used as a control for SLE. Four synovial tissue samples were obtained from three women 53 to 68 years old and a 68-year-old man with RA during their operation of knee joint arthroplasty. All the samples were obtained after the patients gave their informed consent, and this study was approved by the local institutional ethics committee.

### Sample preparation, two-dimensional electrophoresis, and subsequent Western blotting

A synovial tissue sample from a 53-year-old-woman with RA was washed in phosphate-buffered saline (PBS) and then homogenized in a deionized lysis buffer (7 M urea, 2 M thiourea, 4% 3-[(3-Cholamidopropyl)dimethylammonio]-1-propanesulfonate) using HG30 homogenizer (Hitachi Koki Co., Ltd., Tokyo, Japan) on ice. Next, the sample was frozen and thawed five times and then centrifuged at 4°C for 30 minutes. Finally, the supernatant was collected and its protein concentration was determined using the Bradford method. The supernatant was stored at -80°C until use.

The two-dimensional electrophoresis (2DE) was performed as described elsewhere [19,20]. The first electrophoresis is isoelectric focusing (IEF), in which the extracted proteins were loaded onto 11-cm Imobiline drystrip gels (pH range 4 to 7; GE Healthcare, Buckinghamshire, UK) at 20°C for 12 hours. One hundred fifty micrograms of the extracts was applied to the drystrip gels for detection of antigens and 500 μg for identification of proteins by mass spectrometry (MS). IEF was performed using Ettan IPGphor (GE Healthcare Bio-Sciences Corp.). The second electrophoresis was 12.5% SDS-PAGE. After the electrophoresis, the gels were stained with a fluorescent dye of SYPRO Ruby (Molecular Probes, now part of Invitrogen Corporation, Carlsbad, CA, USA) and then used for protein transfer onto nitrocellulose membranes.

Western blotting after 2DE was performed as described previously [2]. Briefly, the proteins transferred onto membranes were reacted with pooled serum samples from five patients with RA or pooled serum samples from five healthy donors to detect autoantigens. The sera were used at a dilution factor of 1:500 per patient. After washing in PBS, bound antibodies were reacted with horseradish peroxidase (HRP)-conjugated goat anti-human IgG (Zymed Laboratories, Inc., now part of Invitrogen Corporation), and were then visualized with diaminobendzidine. Citrullinated proteins on the membranes were detected by Western blotting by using anti-citrulline (modified) detection kit (Upstate Biotechnology, Lake Placid, NY, USA). Specifically, citrulline residues of the proteins immobilized on the membranes were modified by 2, 3-butanedione monoxime and antipyrine in a strong acid solution in accordance with the manufacturer's instructions. Then, the modified citrulline residues were detected by the rabbit polyclonal anti-modified citrulline (anti-MC) antibodies (Upstate Biotechnology) and goat anti-rabbit IgG-HRP antibody conjugate (Upstate Biotechnology). F-actin capping protein α-1 subunit (CapZα-1) was detected by Western blotting using an anti-CapZα-1 polyclonal antibody (Chemicon International, Temecula, CA, USA) and a goat polyclonal anti chicken IgY (H+L)-HRP antibody (Abcam, Cambridge, UK). Rabbit Ig fraction (Dako Denmark A/S, Glostrup, Denmark) was used as a negative control for the anti-MC antibody, and normal chicken IgY (Santa Cruz Biotechnology, Inc., Santa Cruz, CA, USA) was used as a negative control for anti-CapZα-1 antibody.

### Protein identification

Protein spots on the gel stained with SYPRO Ruby which corresponded to the positive spots by Western blotting were recovered and then subjected to in-gel digestion with trypsin. Next, the mass of the digested proteins, extracted by C18 beads, was measured using matrix-assisted laser disorption/ionization-time of flight (MALDI-TOF) MS as described previously [21,22]. Mass spectra of positively charged ions were recorded on a Bruker Ultraflex TOF/TOF instrument (Bruker Daltonik GmbH, Bremen, Germany) operated in the reflector mode. The flexControl, flexAnalysis, and Biotools software packages provided by manufacturer were used for data processing. A list of determined peptide masses were subjected to mass fingerprinting by using the Mascot Search software program (Matrix Science Ltd., London, UK), in which the National Center for Biotechnology Information (NCBI) (Bethesda, MD, USA) protein databases were searched.

### Preparation of recombinant proteins

In accordance with the nucleotide sequence of the human CapZα-1, two DNA primers of 5'-tttccatggccgacttcgatgatcg and 3'-tttctcgagagcattctgcatttctttgccaatc were prepared. A DNA fragment for the entire protein-coding region of CapZα-1 was amplified from cDNA prepared from human synoviocytes by using reverse transcription-polymerase chain reaction. The amplified DNA fragment for CapZα-1 was cloned into a plasmid expression vector of pETBlue-2 (Novagen; Merck KGaA, Darmstadt, Germany) by using restriction endonucleases of Nco I and Xho I. Thereby, recombinant CapZα-1 with a tag of six histidines in its C-terminal was produced in *Escherichia coli *(DE3). The recombinant protein was purified using histidine-Ni^+ ^affinity (His Trap; GE Healthcare Bio-Sciences Corp.) as described previously [23].

### Citrullination of CapZα-1

The recombinant CapZα-1 was citrullinated in several concentrations of rabbit muscle PAD (Sigma-Aldrich, St. Louis, MO, USA). One milligram of the recombinant CapZα-1 was loaded into a Ni^+^-bound column (His Trap). After washing, the column-bound CapZα-1 was reacted with 20 U/mg of PAD in a buffer containing 0.1 M Tris-HCl (pH 7.6), 10 mM CaCl_2_, and 5 mM dithioerythritol and incubated for 2 hours at 37°C. After the second washing, the citrullinated CapZα-1 was eluted by addition of an elution buffer containing 500 mM imidazole. Citrullination of the recombinant CapZα-1 was estimated by Western blotting using an anti-citrulline (modified) detection kit (Upstate Biotechnology).

### Enzyme-linked immunosorbent assay

Enzyme-linked immunosorbent assay (ELISA) was performed as described previously [24]. Briefly, each well of a multititer plate (Immulon; Thermo Labsystems, Franklin, MA, USA) was coated with 2.5 μg/ml CapZα-1 or citrullinated CapZα-1 in a carbonate buffer (50 mM sodium carbonate, pH 9.6). The serum samples diluted at 1:400 were reacted with the immobilized CapZα-1. Then, the bound antibodies were reacted with HRP-conjugated goat anti-human IgG (Invitrogen Corporation). The reactivity of the serum samples in response to the citrullinated or non-citrullinated recombinant CapZα-1 was expressed as optical density (OD) or as arbitrary binding units according to the following formula: sample (binding units) = (OD sample/[mean OD normal sera + 2 standard deviations of normal sera] × 100). According to this formula, 100 binding units was defined as the cutoff point for reactivity.

### Purification of autoantibodies to citrullinated CapZα-1 and subsequent Western blotting

A citrullinated CapZα-1-bound column was created by coupling the citrullinated recombinant CapZα-1 into an *N*-hydroxysuccinimide (NHS)-activated sepharose column (HiTrap NHS-activated HP; GE Healthcare Bio-Sciences Corp.) in accordance with the manufacturer's instructions. Next, a mixture of serum samples from four patients with RA was loaded into the citrullinated CapZα-1-bound column. After washing, autoantibodies against citrullinated CapZα-1 in RA sera were eluted. The concentration of purified antibodies was measured using a human IgG quantitation kit (Bethyl Laboratories, Inc., Montgomery, TX, USA). The purified antibodies and control human IgG (Invitrogen Corporation) diluted to the concentration of 1 μg/ml and the RA serum mixture diluted at 1:500 were reacted with proteins from three synovial samples separated by SDS-PAGE.

### Statistical analysis

Differences of prevalence of the anti-non-citrullinated and citrullinated CapZα-1 antibodies among disease categories were compared using the χ^2 ^test. The differences in mean binding units between the disease categories and in mean clinical parameters between groups were compared by Student's *t *test. The differences in the mean clinical parameter among four groups separated by the patterns of autoantibodies to non-citrullinated and citrullinated CapZα-1 were compared by one-factor analysis of variance or Kruskal-Wallis test. Spearman's correlation coefficient by rank test was used to measure the correlation between titers of antibody and clinical parameters.

## Results

### Detection of citrullinated autoantigens in synovial tissue of a patient with RA

**Figure 1:**
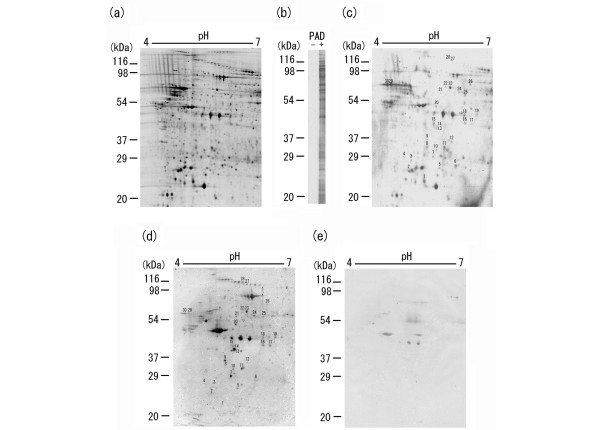
Detection of citrullinated synovial autoantigens in rheumatoid arthritis (RA). Proteins extracted from synovium of a patient with RA were separated by two-dimensional electrophoresis, and protein spots were stained by SYPRO Ruby gel stain **(a)**. Then, the proteins were transferred onto nitrocellulose membranes and reacted with **(c) **anti-modified citrulline (anti-MC) antibodies after modification, **(d) **pooled serum samples from five patients with RA (diluted at 1:500 per person), and **(e) **pooled serum samples from five healthy donors (diluted at 1:500 per person). For these experiments, the capability of the anti-MC antibodies to detect citrullinated proteins was confirmed by response to the cell lysate of *Escherichia coli *treated or not treated with peptidylarginine deiminase (PAD) **(b)**. Protein spots that reacted with the RA sera and the anti-citrulline antibodies, but not with the sera from healthy donors, were thought to be candidates for citrullinated synovial autoantigens, indicated by the numbers 1–30 in **(c) **and **(d)**.

To survey citrullinated autoantigens, we first separated proteins extracted from synovial tissue of a patient with RA by 2DE. Specifically, the proteins were separated by IEF in the range from pH 4 to 7. Then, the separated proteins were further separated by their molecular weights by SDS-PAGE. The separated proteins were stained with a fluorescent dye of SYPRO Ruby by which we detected 990 protein spots (Figure 1a). Then, the proteins were transferred to nitrocellulose membranes and were reacted with the anti-MC antibodies (Figure 1c), pooled sera from five patients with RA (Figure 1d), or pooled sera from five healthy donors (Figure 1e). In addition, to confirm the capability of the anti-MC antibodies to detect citrullinated proteins, we reacted the anti-MC antibodies with cell lysate of *E. coli *(DH5α) with or without citrullination (Figure 1b). As a result, no protein band was detected in the non-treated sample; on the other hand, many protein bands were detected by the anti-MC antibodies in the treated sample. This confirmed that the anti-MC antibodies work well to detect citrullinated proteins. Control rabbit IgG for anti-MC antibodies did not react to any protein spot on 2DE membrane (data not shown). The numbers of protein spots are summarized in Table 1. We found that 51 out of the visualized 990 synovial proteins were citrullinated. This result showed that only a small proportion (5.2%) of synovial proteins were citrullinated. Ninety-four (9.5%) out of the 990 protein spots were reactive to the RA sera but not to the control sera. Interestingly, 30 (31.9%) out of the 94 RA sera-reactive spots were citrullinated proteins and 30 (58.8%) out of the 51 citrullinated proteins were RA sera-reactive. Therefore, we concluded that the autoantigenicity of proteins was associated with citrullination very strongly in RA (*P *= 5.6 × 10^-35^, χ^2 ^test).

### Identification of the citrullinated autoantigens by mass spectroscopy

We next tried to identify the 30 citrullinated autoantigenic protein spots, revealed by comparison of the three panels of Figure 1c–e. Specifically, we recovered peptides from the 30 protein spots after in-gel digestion by trypsin, then measured masses of them by MALDI-TOF MS, and finally searched the NCBI protein database using the obtained MS and MS/MS data and the MASCOT program. We thus identified 13 out of the 30 protein spots successfully. Information on the identified protein spots is summarized in Table 2. Interestingly, spots 27 and 28 were identified as the fibrinogen gamma chain, and similarly, spot 18 was identified as fibrinogen fragment D. Taking them together, 3 (10%) out of the 30 citrullinated autoantigenic protein spots were found to be derivatives of fibrinogen. This is in accordance with the recent reports in which citrullinated fibrinogen was one of the major citrullinated autoantigens in RA [25,26]. Besides fibrinogen, we found asporin, cathepsin D, β-actin, CapZα-1, albumin, histamine receptor, protein disulfide-isomerase (PDI) (Enzyme Commision number (EC) 5. 3. 4. 1.) ER60 precursor, and mitochondrial aldehyde dehydrogenase (ALDH2) as citrullinated autoantigens in RA. To our knowledge, citrullination of all the proteins (except fibrinogen) has not been reported so far. Also, to our knowledge, autoantigenicity of asporin, CapZα-1, histamine receptor, and ALDH2 has not been reported so far.

### Autoantigenicity of citrullinated and non-citrullinated CapZα-1

**Figure 2:**
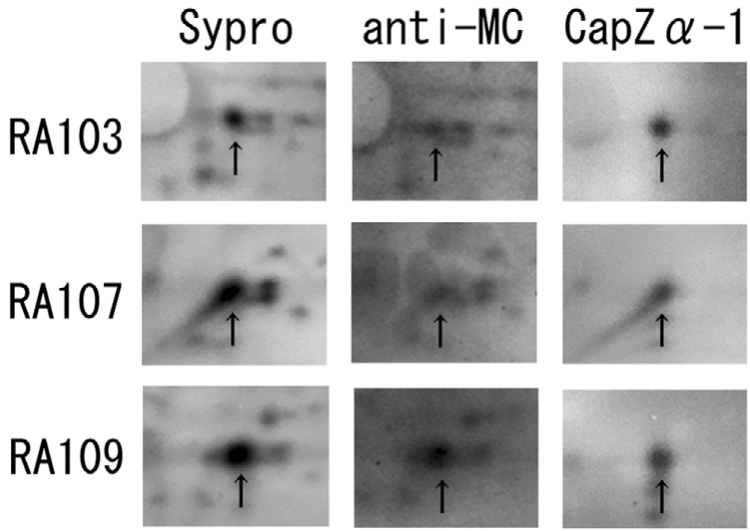
Citrullination of F-actin capping protein α-1 subunit (CapZα-1) in synovial tissues of patients with rheumatoid arthritis. The extracts from synovial tissues from three patients (RA103, 107, and 109) were separated by two-dimensional electrophoresis. Proteins were stained with SYPRO Ruby protein gel stain (left column). After transfer, citrullinated proteins were detected by Western blotting with anti-modified citrulline (anti-MC) antibodies (middle column). The protein spots of CapZα-1 confirmed by Western blotting with anti-CapZα-1 antibodies (right column) are indicated by arrows.

**Figure 3:**
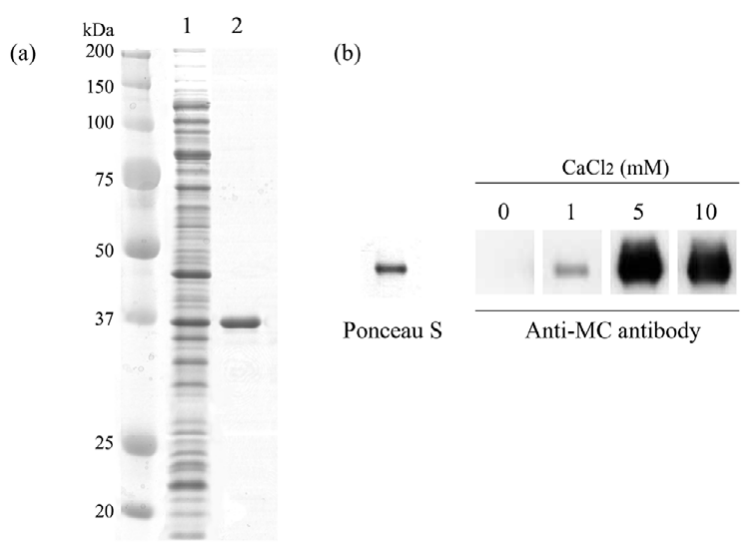
Preparation of recombinant F-actin capping protein α-1 subunit (CapZα-1) with His-tag and citrullination of the recombinant CapZα-1. **(a) **cDNA for the entire protein coding region of human CapZα-1 was amplified by reverse transcription-polymerase chain reaction and then inserted into the pETBlue-2 vector. The full-length CapZα-1 was produced in *Escherichia coli *and purified by using histidine-Ni^+ ^affinity (lane 1, produced CapZα-1 before purification; lane 2, CapZα-1 after purification). **(b) **The recombinant CapZα-1 was reacted with 20 U/mg of peptidylarginine deiminase in various concentrations of CaCl_2_. Then, the citrulline residues were detected by the anti-modified citrulline (anti-MC) antibodies.

We next investigated the contribution of citrullination to autoantigenicity by focusing on CapZα-1, one of the novel citrullinated autoantigens detected. First, to confirm that CapZα-1 in synovial tissue was citrullinated in RA, we separated synovial protein extracts from three more patients with RA by 2DE and checked citrullination of CapZα-1 by the anti-MC antibodies (Figure 2). As a result, CapZα-1 was found citrullinated in all three RA samples. Control chicken IgY for anti-CapZα-1 antibodies did not react to any protein spot on the 2DE membrane (data not shown). Secondly, we prepared a recombinant protein for CapZα-1 by using a bacterial expression system (Figure 3a) and further citrullinated the recombinant CapZα-1 by using PAD. We used rabbit PAD for the citrullination of CapZα-1, in which citrullination occurred depending on concentration of Ca^+ ^as shown in Figure 3b. We confirmed effective citrullination of CapZα-1 with 20 U/mg PAD and 5 to 10 mM Ca^+ ^concentration.

**Figure 4:**
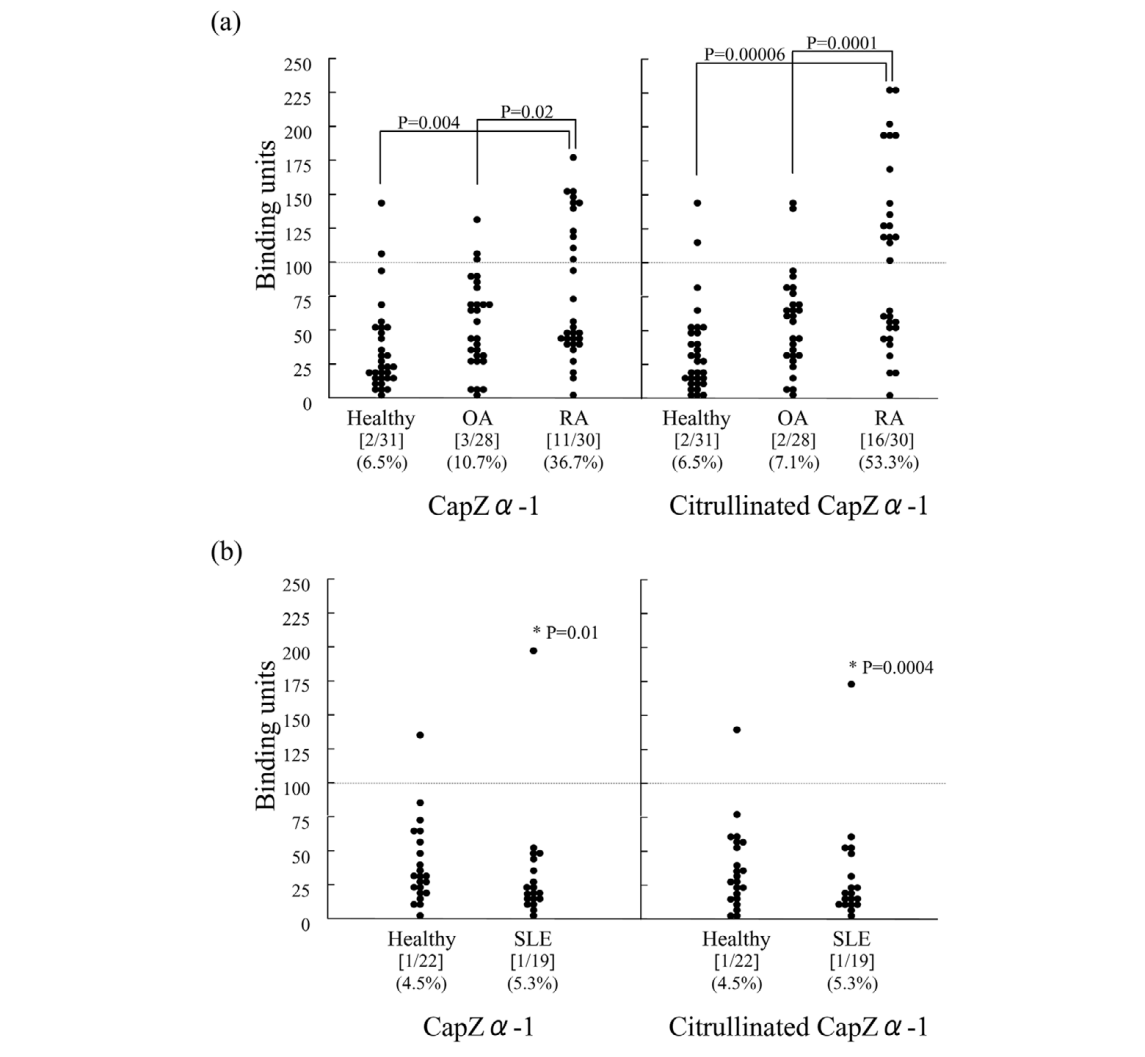
Detection of the autoantibodies to citrullinated or non-citrullinated F-actin capping protein α-1 subunit (CapZα-1) by enzyme-linked immunosorbent assay (ELISA). The autoantibodies to citrullinated or non-citrullinated CapZα-1 were detected by ELISA in patients with rheumatoid arthritis (RA) and osteoarthritis (OA) **(a)**, systemic lupus erythematosus (SLE) **(b)**, and in healthy donors (age- and gender-matched to RA, OA, or SLE groups). The dotted line indicates the cutoff point for positive reaction of 100 binding units. Serum samples diluted at 1:400 were used. The numbers in square brackets indicate the numbers of the antibody-positive serum samples/the numbers of the tested serum samples in each category. The numbers in parenthesis indicate percentages of the antibody-positive serum samples in each category.

Using this recombinant, we tried to determine the frequency of the autoantibodies to CapZα-1 with or without citrullination in patients with RA as well as in patients with OA by ELISA. As a result (Figure 4a), the prevalence of the autoantibodies to CapZα-1 was significantly higher in the RA group (36.7%, 11 out of 30) than in the OA group (10.7%, 3 out of 28) or the healthy group (6.5%, 2 out of 31), even without citrullination of the protein (*P *= 0.02 and *P *= 0.004). Furthermore, it is impressive that the prevalence of the autoantibodies to citrullinated CapZα-1 was elevated to 53.3% only in the patients with RA (16 out of 30), and differences of the frequency between the RA group and the OA group (7.1%, 2 out of 28) and between the RA group and the healthy group (6.5%, 2 out of 31) were more significant statistically (*P *= 0.0001 and *P *= 0.00006). The titers of the autoantibodies to citrullinated CapZα-1 were significantly elevated from those to non-citrullinated CapZα-1 (*P *= 0.003). We further tested the frequency of the autoantibodies in 19 samples of patients with SLE as a non-RA rheumatological control (Figure 4b). As a result, only 1 out of the 19 samples (5.3%) reacted to non-citrullinated CapZα-1, and that sample also reacted to citrullinated CapZα-1. This frequency showed no significant difference compared with the result from healthy donors (4.5%). Differences of the frequency of autoantibodies to non-citrullinated and to citrullinated CapZα-1 between the RA group and the SLE group were statistically significant (*P *= 0.01 and *P *= 0.0004).

### Autoantibodies to citrulline-related epitope(s) and non-related epitope(s) on CapZα-1

**Figure 5:**
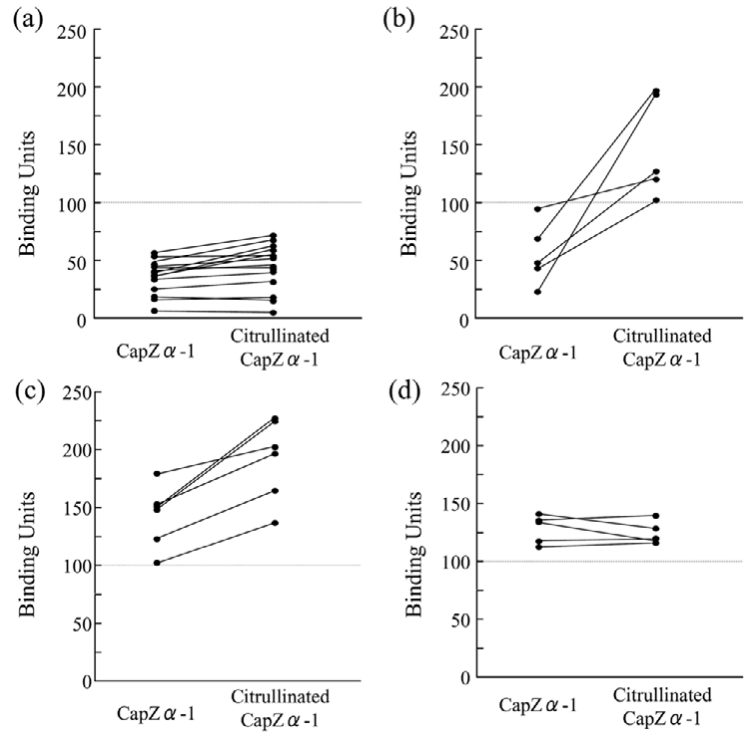
The autoantibody titers to non-citrullinated and citrullinated F-actin capping protein α-1 subunit (CapZα-1) in the individual patients with rheumatoid arthritis. The patients were classified into four groups according to the reactive patterns. **(a) **The autoantibodies both to non-citrullinated and citrullinated CapZα-1 were negative (group A). **(b) **The autoantibodies to non-citrullinated CapZα-1 were negative, and the autoantibodies to citrullinated CapZα-1 were positive (group B). **(c) **The autoantibodies to non-citrullinated CapZα-1 were positive, and the autoantibodies to citrullinated CapZα-1 were further increased (group C). **(d) **The autoantibodies both to non-citrullinated and citrullinated CapZα-1 were positive with similar titers (group D).

We next analyzed differences of the autoantibody titers to non-citrullinated and citrullinated CapZα-1 on each serum sample to evaluate the contribution of citrullination on antigenicity. As shown in Figure 5, the patients with RA were classified into four groups by their reactive patterns. The first group (group A; 46.7%, 14 out of 30) had no autoantibodies to citrullinated or non-citrullinated CapZα-1 (Figure 5a). The second group (group B; 16.7%, 5 out of 30) reacted only to citrullinated CapZα-1 but not to non-citrullinated CapZα-1 (Figure 5b). In this group, citrulline residues would be essential to expression of autoantigenicity of CapZα-1. The third group (group C; 20.0%, 6 out of 30) had positive antibody titers to non-citrullinated CapZα-1 and further increased titers to citrullinated CapZα-1 (Figure 5c). This group was considered to carry both autoantibodies to citrulline-related epitope(s) and citrulline-unrelated epitope(s) on CapZα-1. The last group (group D; 16.7%, 5 out of 30) showed positive antibody titers to citrullinated and non-citrullinated CapZα-1 with similar titers (Figure 5d). This group was concluded to have autoantibodies only to citrulline-unrelated epitope(s). From these data, we concluded that 36.7% (groups B and C; 11 out of 30) of the tested RA patients had autoantibodies to citrulline-related epitope(s) on CapZα-1. On the other hand, no one had autoantibodies to citrulline-related epitope(s) among the healthy donors and only one had autoantibodies to citrulline-related epitope(s) among the patients with OA (data not shown).

### Cross-reactivity of the autoantibodies to citrullinated CapZα-1

**Figure 6:**
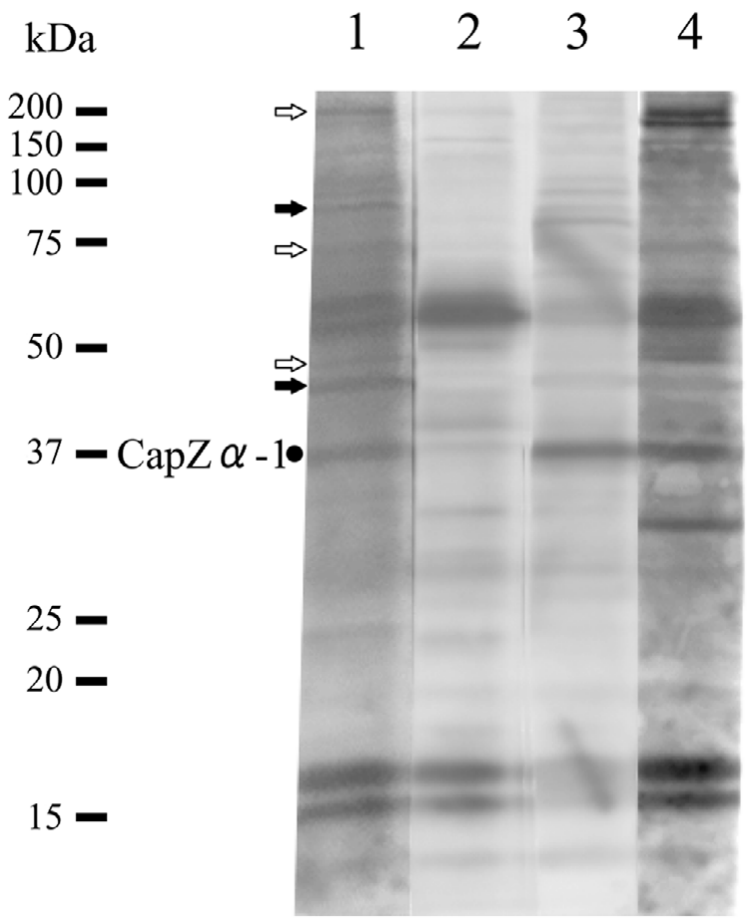
Cross-reactivity of autoantibodies to citrullinated F-actin capping protein α-1 subunit (CapZα-1). To investigate cross-reactivity to other synovial proteins, the autoantibodies purified with citrullinated CapZα-1 (lane 1), control human immunoglobulin G (lane 2), polyclonal anti-CapZα-1 antibodies (lane 3), and rheumatoid arthritis serum samples (lane 4) were reacted with synovial tissue proteins separated by SDS-PAGE. The purified autoantibodies cross-reacted with five protein bands (arrows). Three bands (white arrows) were not reacted with polyclonal anti-CapZα-1 antibodies. On the other hand, two bands (black arrows) were reacted with polyclonal anti-CapZα-1.

To investigate whether the autoantibodies to citrullinated CapZα-1 cross-react to other synovial proteins, we first purified the autoantibodies to citrullinated CapZα-1 from four RA serum samples in groups B and C (Figure 5b,c). Then, the autoantibodies were reacted with synovial tissue proteins separated by SDS-PAGE. As shown in Figure 6, the autoantibodies purified with citrullinated CapZα-1 cross-reacted to five protein bands (indicated by arrows). Three out of the five bands (indicated by white arrows) were not reacted with the polyclonal anti-CapZα-1 antibodies; on the other hand, the remaining two bands (indicated by black arrows) were also reacted with the polyclonal anti-CapZα-1 antibodies. Thus, we conclude that the autoantibodies to citrullinated CapZα-1 have cross-reactivity to at least three other proteins that are not related to CapZα-1.

### Comparison of clinical parameters among the different reactivity groups

We next investigated clinical parameters among the four groups classified above (Table 3). The mean age in group A has a tendency to be lower than other groups and was statistically lower than that of group B (*P *= 0.003). Furthermore, the mean age in groups that did not react to citrulline-related epitope(s) (groups A and D) was significantly lower than that of groups that reacted to citrulline-related epitope(s) (groups B and C) (*P *= 0.02). These findings indicate that autoantigenicity to citrulline-related epitope(s) correlates with age. The mean of anti-CCP antibody titers in group D, which recognized only citrulline-unrelated epitope(s), was significantly higher than those in group A, which did not react to either citrulline-related or citrulline-unrelated epitope(s) (*P *= 0.02), and group C, which reacted to both citrulline-related and -unrelated epitope(s) (*P *= 0.0426). We could not find a definite association of the anti-citrullinated or non-citrullinated CapZα-1 with the anti-CCP antibodies. However, as far as examining the comparison between groups C and D, patients with anti-citrullinated and non-citrullinated CapZα-1 antibodies may have mechanisms to avoid generation of the anti-CCP antibodies. In fact, we found an anti-CCP-negative serum sample in group C, which reacted to citrulline-related epitope(s) on CapZα-1. Studies with greater numbers of patients will be needed.

## Discussion

Here, we examined autoantigenic citrullinated proteins in a synovial tissue sample from a patient with RA by using the proteomic approach and then investigated the contribution of citrullination to autoantigenicity on one of the identified autoantigens, CapZα-1. Our findings are as follows: (a) Out of the 990 synovial tissue protein spots detected by 2DE, 51 protein spots were citrullinated and 94 protein spots were autoantigenic in RA. Thirty protein spots were both citrullinated and autoantigenic. (b) Among the 30 citrullinated and autoantigenic protein spots, 13 protein spots were identified in which derivative peptides of fibrinogen accounted for 3. (c) New identified citrullinated autoantigens were asporin, cathepsin D, β-actin, CapZα-1, albumin, histamine receptor, PDI (EC 5. 3. 4. 1.) ER60 precursor, and ALDH2. (d) In the investigation of autoantigenicity of CapZα-1, citrullination of CapZα-1 was confirmed in all four of the synovial tissue samples from patients with RA. Some patients with RA carried autoantibodies only to citrulline-unrelated epitope(s) on CapZα-1, some carried autoantibodies only to citrulline-related epitope(s) on CapZα-1, and others carried both autoantibodies. (e) Clinically, the patients who had autoantibodies to neither citrullinated CapZα-1 nor non-citrullinated CapZα-1 appeared to be younger than the others. Also, the groups that do not have antibodies to the citrulline-related epitope(s) were significantly younger than the other groups. The anti-CCP antibody titers were not correlated with the titers to CapZα-1.

The first finding indicates that citrullination would be one of the major factors for self-proteins to get autoantigenicity in RA, given that a considerable proportion (30/51, 58.8%) of the citrullinated protein spots were autoreactive. On the other hand, approximately 70% of the autoantigenic protein spots detected were found to be non-citrullinated. Citrullination would not be the only way to get autoantigenicity in RA. The degree of the relation between citrullination and autoantigenicity would differ among different patients with RA. Given that only one synovial sample was available in this screening, larger numbers of RA synovial samples should be investigated in the future to evaluate the relation more precisely.

The second point that 3 out of the 13 identified protein spots were derivative peptides of fibrinogen confirmed the significance of fibrinogen as a major citrullinated autoantigen in RA as reported previously [9]. Our screening revealed that two out of the three fibrinogen peptides were assigned to the fibrinogen gamma chain, not alpha or beta chains (Table 2). The fibrinogen gamma chain may be a major citrullinated autoantigen as well as alpha or beta chains in RA, although the previous study reported that fibrinogen gamma chain was not targeted as frequently as alpha or beta chains were [9]. That could be due to a poor efficiency of citrullination by PAD enzyme *in vitro *as recently described [27].

Besides the fibrinogen chains, we identified several new citrullinated autoantigens successfully. The first is asporin, one of the extracellular matrix components expressed abundantly in the articular cartilage of patients with OA [28]. Asporin is reported to suppress transforming growth factor (TGF)-β-mediated gene expression of aggrecan and type II collagen by inhibiting TGF-β function, possibly through a direct physical interaction with TGF-β [29]. A genetic association with OA of two functional alleles of the asporin gene was reported recently [29]. Thus, citrullination of asporin and binding of the autoantibodies to asporin may alter chondrocyte metabolism in RA. The second is cathepsin D, a lysosomal aspartic peptidase, which is reported to play roles in destroying synovial tissue and cartilage matrix [30,31]. It is interesting whether citrullination affects the functions of cathepsin D. The third is histamine, a classic mediator of inflammation, which was reported to enhance production of matrix metalloprotease-1 by rheumatoid synovial fibroblasts via H1 receptors and to enhance interleukin (IL)-1-α-induced IL-6 and IL-1-β synthesis by peripheral blood mononuclear cells via H2 receptors [32-34]. It is also interesting whether citrullination of histamine receptors and/or binding of autoantibodies to histamine receptors affects the inflammatory conditions in RA.

In addition to the three molecules, we identified β-actin, albumin, and PDI ER60 precursor. PDI ER60 precursor was thought to be a thiol protease. Autoantibodies to β-actin were reported in patients with autoimmune inner ear disease [35,36]. Autoantibodies to *N*-homocysteinylated albumin have been reported as a marker for coronary artery disease [37]. Autoantibodies to PDI were detected in a spontaneous rat hepatitis model and in patients with SLE, infertility, or allergic rhinitis [38-41]. However, function of the autoantibodies remains to be solved in these diseases as well as in RA. Similarly, effects of citrullination on the functions of these molecules should be investigated. Our screening did not identify some of the major known citrullinated autoantigens such as EBVA-1 and vimentin. A single screening is not ideal for identifying all the citrullinated proteins. Repeated screening would elucidate greater numbers of citrullinated proteins.

In the investigation of autoantigenicity of CapZα-1, citrullination of CapZα-1 was confirmed in all four of the tested synovial tissue samples from the patients with RA. The frequency of the autoantibodies to non-citrullinated CapZα-1 was significantly higher in the RA group than in the OA, SLE, and healthy groups. Further, the frequency and titers of the autoantibodies to CapZα-1 increased when citrullinated CapZα-1 was used as an antigen only in the RA group. Therefore, both of the autoantibodies to citrulline-related and -unrelated epitope(s) on CapZα-1 would be closely related to RA. However, it appears to depend on individual patients whether they carry autoantibodies to the citrulline-related epitope(s), to the citrulline-unrelated epitope(s), or to both epitope(s). The autoantibodies purified by the citrullinated CapZα-1 showed cross-reactivity to other proteins, and we detected five protein bands reacted with the antibodies. Two of the five protein bands reacted with the polyclonal anti-CapZα-1 antibodies, indicating existence of similar epitope(s) to non-citrullinated CapZα-1. The remaining three would have other epitope(s) similar to citrullinated parts of CapZα-1. The titers of the anti-CCP antibodies were significantly higher in the patients with antibodies to citrulline-unrelated epitope(s) (group D) than those in the patients with only antibodies to citrulline-related epitope(s) (group C). None of the sera of patients with OA or of healthy donors with reactivity to citrullinated CapZα-1 reacted with CCP. Only one patient each in the OA group and the healthy group had anti-CCP antibodies, but they did not have anti-citrullinated CapZα-1 antibodies (data not shown). These results indicate that the titers of the anti-CCP antibodies do not always represent total antibodies to citrullinated epitopes.

Clinically, the mean age of the groups that carry autoantibodies to citrulline-related epitope(s) (groups B and C) was significantly higher than that of the other groups (groups A and D). Further, in the groups not having antibodies to non-citrullinated CapZα-1, the mean age of the group that carries autoantibodies to citrulline-related epitope(s) (group B) is significantly higher than that of the group that carries antibodies neither to citrulline-related nor-unrelated epitope(s) (group A). The differences of the means of duration were not significant among them. Because the number of patients tested is rather small, we need further investigation with larger numbers of samples to clarify the association with clinical data.

F-actin capping protein (CapZ), a heterodimeric protein consisting of subunits of α and β that bind selectively and with high affinity to the barbed ends of actin filaments [42], is present in a wide variety of tissues and organisms. Vertebrates, including chicken, mice, and humans, contain two α-subunit isoforms and two β-subunit isoforms [43,44]. The α1 and α2 isoforms share 85% amino acid identity [45,46]. CapZ regulates the growth of actin filament by capping the barbed ends of actin filaments [42]. Previous studies suggest that CapZ is essential to the assembly and organization of the actin cytoskeleton *in vivo *[47-50]. Therefore, citrullination of CapZα-1 may inhibit the activity of capping protein and can lead to degeneration of various cells such as synoviocytes and chondrocytes, resulting in the release of antigens to inflammatory cells. Further studies are needed to elucidate whether the pathway is possible.

In summary, we demonstrated a profile of citrullinated autoantigens in RA synovium. Autoantigenicity and citrullination are closely related, but citrullination does not entirely explain autoantigenicity in RA. Our data will help in understanding the roles of citrullination in RA.

## Abbreviations

ALDH2 = mitochondrial aldehyde dehydrogenase; anti-MC = anti-modified citrulline; CapZα-1 = F-actin capping protein α-1 subunit; CCP = cyclic citrullinated peptide; EBVA-1 = Epstein-Barr virus nuclear antigen 1; EC = enzyme commission number; ELISA = enzyme-linked immunosorbent assay; HRP = horseradish peroxidase; IEF = isoelectric focusing; Ig = immunoglobulin; IL = interleukin; MALDI-TOF = matrix-assisted laser disorption/ionization-time of flight; MS = mass spectrometry; NCBI = National Center for Biotechnology Information; OA = osteoarthritis; OD = optical density; PAD = peptidylarginine deiminase; PADI4 = peptidylarginine deiminase-4; PBS = phosphate-buffered saline; PDI = protein disulfide-isomerase; RA = rheumatoid arthritis; SF = synovial fluid; SLE = systemic lupus erythematosus; TGF = transforming growth factor; 2DE = two-dimensional electrophoresis.

## Competing interests

The authors declare that they have no competing interests.

## Authors' contributions

K Matsuo carried out the molecular biological studies and drafted the manuscript. YX helped to carry out the immunoassays. HN, K Masuko, KY, K Noyori, K Nishioka, and TS participated in the design of the study and performed the statistical analysis. TK conceived of the study, participated in its design and coordination, and helped to draft the manuscript. All authors read and approved the final manuscript.

## Figures and Tables

**Table 1:** Numbers of citrullinated and/or autoreactive synovial protein spots

		Rheumatoid arthritis sera		Total
		Reactive	Non-reactive	
Anti-citrulline	Reactive	30^a^	21	51
	Non-reactive	64	875	939
Total		94	896	990

^a^*P *= 5.6 × 10^-35^.

**Table 2:** Citrullinated autoantigens identified using mass spectrometry

Spot number	Observed		Protein name	Mascot score	Number of peptides	Accession number	Calculated	
						
	pI	MW (kDa)					pI	MW (kDa)
1	5.4	21	n.d.					
2	5.1	24	n.d.					
3	5.15	28	n.d.					
4	5.0	30	n.d.					
5	5.75	25	n.d.					
6	6.1	26	n.d.					
7	5.7	31	Mutant β-actin	101	1	gi| 28336	5.22	42.1
8	5.5	34	n.d.					
9	5.5	36	n.d.					
10	5.7	33	n.d.					
11	5.8	33	Cathepsin D, chain B	120	8	gi| 494296	5.31	26.5
12	6.0	35	n.d.					
13	5.7	40	n.d.					
14	5.7	41	n.d.					
15	5.6	41	F-actin capping protein α-1 subunit (CapZα-1)	85	6	gi| 5453597	5.45	33.1
16	6.25	44	Asporin precursor Asporin Periodontal ligament associated protein-1	81 81 81	1 1 1	gi|13625797 gi|41350214 gi|16151085	6.84 5.36 6.43	43.7 43.8 43.9
17	6.4	43	Asporin precursor	36	1	gi| 13625797	6.84	43.7
18	6.25	47	Chain B, crystal structure of fibrinogen fragment D	75	9	gi| 2781208	5.85	37.6
19	6.5	48	Asporin precursor	65	8	gi| 13625797	6.84	43.7
20	5.7	51	Mutant β-actin 48-kDa histamine receptor subunit peptide-4 (internal fragment)	62 62	1 1	gi| 28336 gi| 998467	5.22 4.41	42.1 31.4
21	5.75	58	n.d.					
22	5.8	61	n.d.					
23	5.95	61	Protein disulfide-isomerase (EC 5. 3. 4. 1.) ER60 precursor Glucose-related protein 58 kDa	201 201	20 20	gi|1085373 gi|27805905	6.10 6.23	56.6 56.9
24	6.1	59	Chain A, human mitochondrial aldehyde dehydrogenase complexed with NAD^+ ^and Mn^2+^	122	2	gi| 6137677	5.70	53.9
25	6.3	58	n.d.					
26	6.4	67	Human serum albumin in a complex with myristic acid and tri-iodobenzoic acid Chain A, crystal structure of human serum albumin	83 82	9 9	gi|4389275 gi|3212456	5.69 5.67	66.0 66.4
27	5.85	102	Recombinant human gamma-fibrinogen carboxyl terminal fragment	63	6	gi| 2554640	5.87	28.2
28	5.75	102	Fibrinogen gamma	102	7	gi| 223170	5.54	46.3
29	4.7	62	n.d.					
30	4.8	62	n.d.					

MW, molecular weight; n.d., not determined; pI, isoelectric point.

**Table 3:** Comparison of clinical data among groups A-D, between antibodies to citrulline-related epitope (positive and negative), and between antibodies non-related epitope (positive and negative)

Group		Number of patients	Age in years	Duration	Female/Male	RF	CCP	CRP	ESR
A		14	56.1 ± 10.8^a^	12.2 ± 9.2	12/2	89.6 ± 116.7	63.3 ± 66.9^c^	1.55 ± 1.86	33.2 ± 16.8
B		5	66.6 ± 2.1^a^	11.4 ± 5.4	4/1	422.6 ± 574.5	385.6 ± 396.1	0.88 ± 0.79	41.8 ± 23.4
C		6	65.7 ± 7.8	17.2 ± 12.7	6/0	139.3 ± 146.2	63.8 ± 55.5^d^	0.97 ± 0.95	38.3 ± 27.9
D		5	61.2 ± 21.0	6.3 ± 5.8	4/1	47.5 ± 47.0	164.0 ± 84.9^c, d^	1.18 ± 1.48	31.0 ± 19.1
Anti-citrullinated CapZα-1	+(B+C+D)	16	64.6 ± 12.0	11.6 ± 9.2	14/2	199.2 ± 348.5	195.7 ± 253.2	1.01 ± 1.03	37.1 ± 22.9
	-(A)	14	56.1 ± 10.8	12.2 ± 9.2	12/2	89.6 ± 116.7	63.3 ± 66.9	1.55 ± 1.86	33.2 ± 16.8
Citrulline-unrelated epitope	+ (C+D)	11	63.6 ± 14.6	11.7 ± 10.9	10/1	97.6 ± 117.8	109.3 ± 84.7	1.06 ± 1.16	35.0 ± 23.4
	-(A+B)	19	58.8 ± 10.4	12.0 ± 8.2	16/3	177.3 ± 325.4	148.1 ± 243.7	1.37 ± 1.65	35.5 ± 18.5
Citrulline-related epitope	+ (B+C)	11	66.1 ± 5.7^b^	14.3 ± 9.7	10/1	268.1 ± 405.7	210.1 ± 304.2	0.93 ± 0.84	39.9 ± 24.7
	-(A+D)	19	57.4 ± 13.7^b^	10.7 ± 8.7	16/3	78.6 ± 103.4	89.8 ± 83.2	1.45 ± 1.74	32.6 ± 16.9

^a^*P *< 0.005; ^b,c,d^*P *< 0.05. CapZα-1, F-actin capping protein α-1 subunit; CCP, cyclic citrullinated peptide; CRP, C-reactive protein; ESR, erythrocyte sedimentation rate; RF, rheumatoid factor.
